# Exogenous Administration of a Recombinant Variant of TWEAK Impairs Healing after Myocardial Infarction by Aggravation of Inflammation

**DOI:** 10.1371/journal.pone.0078938

**Published:** 2013-11-11

**Authors:** Christina Pachel, Denise Mathes, Barbara Bayer, Charlotte Dienesch, Gaby Wangorsch, Wolfram Heitzmann, Isabell Lang, Hossein Ardehali, Georg Ertl, Thomas Dandekar, Harald Wajant, Stefan Frantz

**Affiliations:** 1 Department of Internal Medicine I, University Hospital Würzburg, Würzburg, Germany; 2 Comprehensive Heart Failure Center, University of Würzburg, Würzburg, Germany; 3 Department of Bioinformatics, Biocenter, University of Würzburg, Würzburg, Germany; 4 Division of Molecular Internal Medicine, Department of Internal Medicine II, University Hospital Würzburg, Würzburg, Germany; 5 Feinberg Cardiovascular Research Institute, Northwestern University, Chicago, Illinois, United States of America; Albert Einstein College of Medicine, United States of America

## Abstract

**Background:**

Tumor necrosis factor-like weak inducer of apoptosis (TWEAK) and its receptor fibroblast growth factor-inducible 14 (Fn14) are upregulated after myocardial infarction (MI) in both humans and mice. They modulate inflammation and the extracellular matrix, and could therefore be important for healing and remodeling after MI. However, the function of TWEAK after MI remains poorly defined.

**Methods and results:**

Following ligation of the left coronary artery, mice were injected twice per week with a recombinant human serum albumin conjugated variant of TWEAK (HSA-Flag-TWEAK), mimicking the activity of soluble TWEAK. Treatment with HSA-Flag-TWEAK resulted in significantly increased mortality in comparison to the placebo group due to myocardial rupture. Infarct size, extracellular matrix remodeling, and apoptosis rates were not different after MI. However, HSA-Flag-TWEAK treatment increased infiltration of proinflammatory cells into the myocardium. Accordingly, depletion of neutrophils prevented cardiac ruptures without modulating all-cause mortality.

**Conclusion:**

Treatment of mice with HSA-Flag-TWEAK induces myocardial healing defects after experimental MI. This is mediated by an exaggerated neutrophil infiltration into the myocardium.

## Introduction

Heart failure (HF) is a major cause of mortality in industrialized countries affecting approximately 1–2% of the adult population [1]. The most common reason of HF is an incompletely reperfused myocardial infarction (MI) with subsequent maladaptive left ventricular (LV) remodeling. The immune system plays a major role in the reparative/remodeling response following ischemic injury. It is activated early and subsequently clears cellular debris. In the sub-acute granulation or healing phase, inflammatory cells are necessary for the development of a solid scar [2]. Therefore, in the first phases after MI inflammation is a prerequisite for proper healing. However, in the final remodeling phase, it is important that inflammation is downregulated. A chronic elevation of intramyocardial proinflammatory cytokines, including interleukin (IL)-6 [3] and tumor necrosis factor (TNF) α [4], are associated with adverse cardiac remodeling.

TWEAK is a member of the TNF ligand family and is up-regulated after MI [5]. It is initially expressed as a type II transmembrane protein but typically processed with high efficacy by furin-like proteases to a soluble form [6]. TWEAK functions mainly by binding to its receptor fibroblast growth factor-inducible molecule 14 (Fn14), which has the potential to activate both the classical and alternative nuclear factor-κB (NFκB) [7] as well as various MAPK pathways [8]. It is involved in cell proliferation, -differentiation, apoptosis, angiogenesis, and inflammation [9]. TWEAK exists in two forms: 1) the initially expressed membrane bound form that efficiently triggers all known Fn14-related signaling events including the classical NFκB pathway, and 2) the processed soluble form that activates the alternative NFκB pathway and enhances TNF-induced cell death. The latter form only shows limited pro-inflammatory activities via the classical NFκB pathway and MAP kinases [7], [10].

The function of TWEAK in cardiovascular diseases is controversial. On the one hand, overexpression of full length, thus membrane-bound TWEAK causes dilated cardiomyopathy and cardiac dysfunction in mice. This effect is mediated exclusively by Fn14 receptor and is associated with cardiomyocyte elongation and cardiac fibrosis but not with cardiomyocyte apoptosis [11]. On the other hand, exogenous application of the soluble TWEAK induced hypoxic and ischemic tolerance *in vitro* and *in vivo* in mouse models of cerebral ischemia and decreased the volume of ischemic lesions after transient middle cerebral artery occlusion in an Fn14-dependant manner. This effect was mediated by TNFα and ERK 1/2 activation via phosphorylation of BAD [12]. The divergent findings after ischemia might be due to different effects of the TWEAK forms. Cardiac overexpression of a full length TWEAK resembles the membrane bound form and therefore triggers classical NFκB-signaling, a pathway known to be maladaptive after cardiac ischemia [13], [14].

Due to its immunomodulatory effects, its role in healing, and ischemic protection after stroke we hypothesized that in contrast to membrane bound TWEAK soluble TWEAK could be a cardioprotective target after MI. Thus, we applied a genetically engineered construct after MI that contains soluble form of TWEAK attached to serum albumin domain (HSA-Flag-TWEAK). This chimeric protein possesses an improved serum half-life compared to conventional soluble TWEAK [7].

## Materials and Methods

### Ethics Statement

This study conforms to the “Guide for the Care and Use of Laboratory Animals” published by the US National Institutes of Health. All experiments were performed according to the German regulations for animal experimentation. The study was approved by the Regierung von Unterfranken as the responsible authority (Permit Number 55.2-2531.01-01/10). All surgery was performed under isoflurane or tribromoethanol anesthesia, and all efforts were made to minimize suffering.

### Animals

Eight- to ten-week-old male C57BL/6J mice were obtained from Harlan Laboratories (Eystrup, Germany) and were randomized into two treatment groups: HSA-Flag-TWEAK and placebo. The recombinant protein was produced as described previously [15].

Animals had free access to standard chow and drinking water and were kept under specific pathogen-free conditions.

### Isolation of Primary Cardiac Mouse Fibroblast and Myocytes

Left ventricular tissue from healthy mice was minced into small pieces, digested as described and transferred into tissue culture flasks in Dulbecco’s modified eagle media (DMEM, Lonza, Cologne, Germany) supplemented with 10% fetal bovine serum [16]. For the isolation of primary adult mouse cardiac myocytes the heart was first perfused with calcium-free buffer, digested as described and perfused with calcium buffer [17]. Cardiomyocytes were cultured in Modified Eagle’s Medium (MEM), supplemented with 10% bovine serum albumin.

### Experimental MI

MI was induced in mice as described previously [18]. In brief, mice were anesthetized with isoflurane (Abbott GmbH & Co. KG, Wiesbaden, Germany) intubated, and put on a mechanical small-animal ventilator (MiniVent, Hugo Sachs Elektronik, March-Hugstetten, Germany). After left-sided thoracotomy, ligation of the left coronary artery with a PE-10 tube on top of the vessel was performed with a 6-0 silk suture, 2–3 mm from the tip of the left auricle. The chest wall was closed with a continuous 6-0 prolene suture and the skin with a 4-0 polyester suture. In sham-operated mice, a thoracotomy was performed to expose the heart, but the coronary artery was not ligated. Mice were injected intraperitoneally (i.p.) with HSA-Flag-TWEAK (32 µg/mouse) or placebo control (PBS, 200 µl/mouse) right after inducing myocardial infarction. The optimal HSA-Flag-TWEAK dose was previously determined by us [15], [19]. Treatment with TWEAK or placebo control was repeated six times for 3 weeks. Animals were sacrificed 8 weeks after MI.

### Detection of Cardiac Rupture

Autopsy was performed on all animals either found dead or sacrificed at the end of the study period. Cardiac rupture was confirmed by the presence of clotted blood in the chest and perforation of the infarcted wall, as described previously [20].

### Echocardiography

Ultrasound analyses (Toshiba Aplio system, Neuss, Germany) were performed by a single researcher experienced in rodent echocardiography at day 1, 21 and 56 after MI as described before [18]. For the procedure, mice were put under light general anesthesia with isoflurane to allow for spontaneous respiration. From two-dimensional short axis imaging, endocardial borders were traced at end-systole and end-diastole utilizing a prototype off-line analysis system (NICE, Toshiba Medical Systems, Zoetermeer, Netherlands). Measurements were performed at the mid-papillary muscle level. The end-systolic (smallest) and end-diastolic (largest) cavity areas were determined. Using the end-systolic and -diastolic areas, fractional area changes were calculated [(end-diastolic area - end-systolic area)/end-diastolic area]. From two-dimensionally targeted M-mode tracings, end-diastolic diameter and end-systolic diameter were measured. Fractional shortening was calculated.

### Histology and Immunohistology

Sections of mouse myocardium were embedded in Tissue Tek (Sakura, Alphen aan den Rijn, Netherlands) and 7-µm cryosections were prepared. Cells were labelled with mouse-anti-mouse Fn14 (clone ITEM-4; eBioscience, Frankfurt, Germany), rat-anti-mouse neutrophils (clone 7/4; Linaris, Germany), or rabbi-anti-mouse periostin (BioVendor, Heidelberg, Germany). For the labelling of cleaved PARP, formalin fixed sections of mouse myocardium were prepared in the standard manner. Sections were treated with an anti-peroxidase complex and histoprime enhancer (Linaris, Dossenheim, Germany) before the application of the primary rabbit-anti-mouse cleaved PARP antibody (abcam, Cambridge, United Kingdom). The secondary antibodies were Alexa Fluor 647 goat anti-rabbit IgG (invitrogen), Alexa Fluor 647 goat anti-mouse IgG (invitrogen) and Alexa Fluor 350 goat anti-rat IgG (invitrogen). The slides were embedded in mounting medium (Vector Labs, Peterborough, United Kingdom). Images were obtained with an AxioCamMR3 camera, mounted on an Axio Imager.Z1 microscope (Carl Zeiss, Jena, Germany) equipped with AxioVision software (version 4.8.3.0). 7-µm sections were stained according to a standard hematoxylin-and-eosin staining protocol and embedded in Entellan (Merck, Darmstadt, Germany) for light microscopy (Axioskop 2 plus, Carl Zeiss, Jena, Germany; software: SPOT Basic, version 5.0). For the determination of collagen content, collagen was stained with picrosirius red (picrosirius red F3BA, Polysciences Inc., Warren, Pennsylvania, USA) on 5-µm sections, as described recently [18]. The slides were washed with phosphate buffered saline (PBS) and embedded in Entellan.

### Real-Time Reverse Transcriptase–polymerase Chain Reaction

Total RNA was isolated using the RNeasy Mini kit (Qiagen, Hilden, Germany) and cDNA reverse transcription was performed with iScript cDNA synthesis kit (BioRad, München, Germany) according to manufacturer’s instructions. Real-time polymerase chain reaction (PCR) procedures were performed with commercially available TaqMan probes (Applied Biosystems, Bedford, USA) for GAPDH, TWEAK, Fn14, MMP2, MMP3, MMP8, MMP9, collagen1α1, collagen1α2, TIMP2, TIMP3, and VEGF as reported previously [18]. Target gene ratios were normalized to GAPDH.

### Infarct Size Measurement after Myocardial Infarction

Infarct size measurements after 6 hours of MI were performed as recently described [21]. In short, animals were put under general anesthesia with isoflurane and intubated using a ventilator. The chest was opened and 5% Evans blue (Sigma-Aldrich, Munich, Germany) in PBS was injected into the apex of the heart. After the animals were sacrificed by intracardiac injection of saturated KCl solution in PBS, the heart was removed, washed with 0.9% NaCl and frozen in Tissue Tek (Sakura, Alphen aan den Rijn, Netherlands) at −20°C for 30 minutes. The frozen heart was cut into five parallel transverse slices, which were stained with 2% 2,3,5-Triphenyl-tetrazolium chloride (TTC) (Sigma-Aldrich, Munich, Germany) for 10 min at 37°C. After TTC staining, viable myocardium stains red and the infarcted areas appear pale. Slices were weighed, imaged and the area of infarction for each section was determined by computerized planimetry using an image analysis software program (VGA Planimetrie 2010 for Canon EOS5D and Planimetrie Report for Microsoft Excel 2010).

### Cytokine Arrays

Cytokine levels within the myocardium of placebo or HSA-Flag-TWEAK treated animals were analyzed 3 days after myocardial infarction using Quantibody mouse cytokine array Q2000 (RayBiotech, Norcross GA, USA) according to the manufacturer’s protocol. Standard curves of the cytokines were generated using Origin 8.5.1 (OriginLab, Northampton, USA) following background subtraction.

### Bioinformatic Gene Expression Analysis based on Cytokine Array Results

Gene expression data were normalized and significantly higher expressed genes (ANOVA t-test) were mapped on the interactome. The human interactome network including kinases and phosphorylation substrate information was established for the cardiomyocyte as described previously for the platelet [22]. Briefly, different proteome and transcriptome databases were collated regarding protein nodes with expression evidence in the cardiomyocyte. Information on human protein-protein interactions (PPIs) was obtained from the Human Protein Reference Database (HPRD; version 9.0, April 2010) [23] and the Entrez Gene National Center for Biotechnology Information (NCBI) server [24] and visualized with Cytoscape (version 2.8.3). It was combined with data on protein phosphorylation from HPRD (version 9.0) and PhosphoSite as well as kinase predictions for phosphoproteome data using the NetworKIN algorithm [25], [26]. Furthermore, interaction predictions were generated from a number of protein-protein interaction databases such as HPRD. Gene and protein names were taken according to RefSeq records. A comprehensive list of human kinases was extracted from Manning et al. (2002) and used for reference and validation of the HPRD phosphorylation data [27].

### Gelatine Zymography

Zymography, based on a SDS-PAGE method, was performed to determine colagenolytic activities of MMP-2 and MMP-9 in homogenized heart tissue of placebo- and HSA-Flag-TWEAK-treated WT mice. For protein isolation, myocardium tissue of the infarcted area was homogenized with Ripa buffer and PMSF (Cell Signaling, Frankfurt, Germany). The samples were separated on a 10% polyacrylamide gel containing 2.5 mg/ml gelatine at a constant voltage of 120 V for 2 h at 4°C. After electrophoresis, the proteins were renaturated by incubation of the gels in 2.5% Triton X-100 (Sigma-Aldrich) for 90 min at room temperature. The gels were then incubated in activation buffer (50 mM Tris-HCl, pH 7.5, 5 mM CaCl_2_, 0.2 M NaCl, and 0.02% Brij-35) for 12 h at 37°C. Finally, the gels were stained for 1 h at RT using 0.5% coomassie blue staining solution and then destained in 40% v/v methanol, 10% v/v acetic acid to reveal bands of clearing which indicate proteolytic activity. The band intensity was quantified using ImageJ (version 1.44p).

### Terminal Deoxynucleotidyl Transferase-mediated dUTP Nick-end Labeling (TUNEL) Assay

Nuclear DNA fragmentation (In Situ Cell Death Detection Kit, Fluorescein, Roche Diagnostics, Mannheim, Germany) was used as a marker for apoptosis. Sections of mouse myocardium were fixed in 4% paraformaldehyde overnight, embedded in paraffin, sectioned, and stained according to the manufacturer’s instructions. TUNEL-positive cells were counted in the border zone area - defined as the zone bordering the infarct where viable myocardium was prevalent and reparative fibrosis was only marginal - and in the remote unaffected myocardium (septum region).

### Fluorescence-Activated Cell Sorting (FACS)

For the quantification of infiltrating immune cells into mouse hearts, the monoclonal antibody anti-mouse CD45 eFluor450 (eBioscience, Frankfurt, Germany) was used. For the exclusion of dead cells 7-Amino-Actinomycin D (7-AAD) was used (BD Pharmingen).

Hearts were harvested, the ischemic parts were isolated, and the tissue was digested in a suspension of collagenase (100 mg/ml; Sigma-Aldrich, Schnelldorf, Germany) and protease XIV (protease from Streptomcyces griseus, 50 µg/ml; Sigma-Aldrich) in PBS with calcium and magnesium for 30 minutes at 37°C. A single cell suspension was prepared by filtering the tissue through a 40-µm cell strainer (BD Falcon, Heidelberg, Germany). 10^6^ cells per staining were washed with 100 µl FACS buffer (0.1% BSA, 0.02% NaN_3_ in PBS) once. To block unspecific Fcγ RIII/II receptors, cells were incubated with saturating amounts of cell culture supernatant of the clone 2.4G2 for 15 minutes (4°C). Surface antigens were stained for 15 minutes and cells were washed with FACS buffer once.

For quantification of neutrophils in whole blood samples, the following antibodies were used: anti-mouse CD45 eFluor450 (eBioscience), PE rat anti-mouse CD11b and Alexa Fluor 647 anti-mouse Ly6G (BD Pharmingen). Coagulation of whole blood was averted by heparinization. Following staining of surface antigens, erythrocytes were lysed by incubation with fixation permeabilization concentrate (eBioscience) in fixation permeabilization diluent (eBioscience) for 45 minutes at room temperature.

Cells were analysed in FACS buffer on a BD FACSCanto II flow cytometer (BD, Heidelberg, Germany). Data was analysed with FlowJo (TreeStar Inc) software (version 7.6.5).

### Neutrophil Depletion Study

Mice were injected i.p. with HSA-Flag-TWEAK (32 µg/mouse) or placebo control (PBS) right after inducing myocardial infarction and on days 3 and 6 after MI. Neutrophil granulocytes were depleted in placebo and HSA-Flag-TWEAK-treated animals by i.p. administration of 250 µg of an anti-Ly6G antibody (clone 1A8; Biolegend, London, United Kingdom) or istotype control (clone RTK2758; Biolegend) one day before infarct surgery (day −1) and on days 5 and 7. On day 8 after MI, animals were sacrificed, peripheral blood samples were taken for FACS analysis of neutrophils, and sections of mouse myocardium were fixed in 4% paraformaldehyde overnight, embedded in paraffin, and stained for neutrophils.

### Statistical Analysis

All data are expressed as mean and standard error of mean. Mortality data were compared using a log rank test. Statistical analysis was performed using GraphPad Instat3 software (version 3.0; La Jolla, USA). Treatment groups were compared to the placebo group using two-tailed unpaired student’s t test. Statistical significance was achieved when two-tailed p<0.05.

## Results

### TWEAK and Fn14 are Upregulated after MI

**Figure 1:**
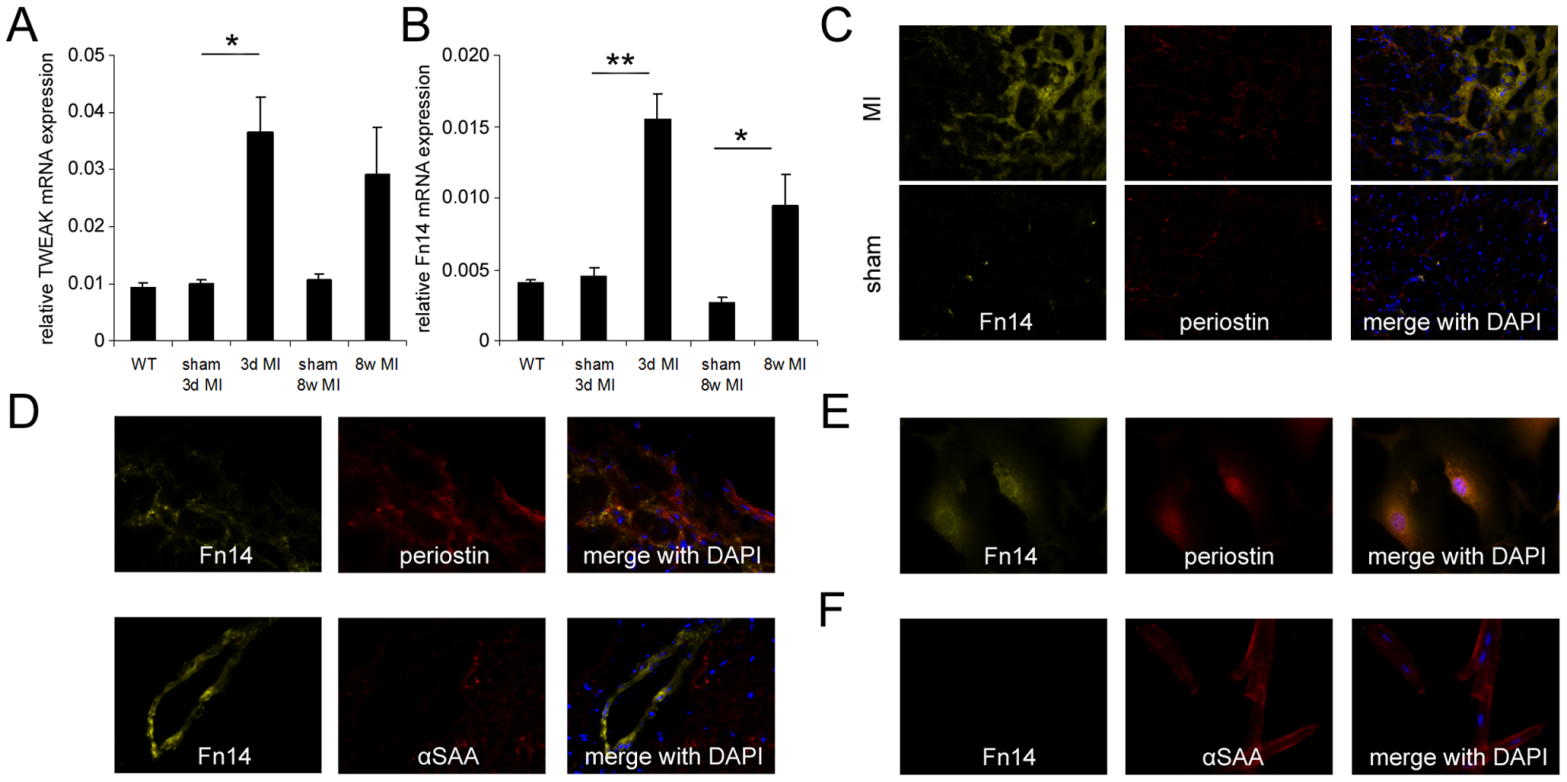
MI induces the expression of TWEAK and Fn14 in the mouse heart. (A) TWEAK and (B) Fn14 mRNA expression were significantly increased 3 days after experimental MI. Fn14 mRNA expression was significantly increased after 8 weeks of MI. (C) Immunohistological staining after 3 day of MI revealed high expression of Fn14 protein in the border zone of the myocardium co-localizing with periostin expression. (D) Periostin positive fibroblasts are the main source of Fn14 expression in the infarcted heart and αSAA positive cardiomyocytes show no expression of Fn14. (E) Isolated cardiac mouse fibroblasts express Fn14 whereas (F) isolated cardiomyocytes show no Fn14 expression.

We analyzed mRNA expression of TWEAK and its receptor Fn14 in mouse heart samples 3 days and 56 days after MI. TWEAK was significantly upregulated 3 days after MI compared to sham operated animals (figure 1A). Fn14 was also significantly upregulated 3 days and 56 days after MI (figure 1B). Immunohistological staining revealed increased expression of Fn14 in the border zone 3 days after MI in comparison to sham operated mice (figure 1C), which was mainly confined to cardiac fibroblasts (figure 1D). These findings could be verified with isolated adult mouse cardiomyocytes and fibroblasts (figure 1E, 1F) with only the latter cells expressing Fn14. These results indicate that both TWEAK and its receptor Fn14 are upregulated after MI in mice and increased Fn14 expression is mainly confined to fibroblasts, suggesting an involvement of the TWEAK-Fn14 axis in cardiac wound healing.

### Exogenous Soluble TWEAK Increases Mortality after MI and is Associated with Cardiac Ruptures

**Figure 2:**
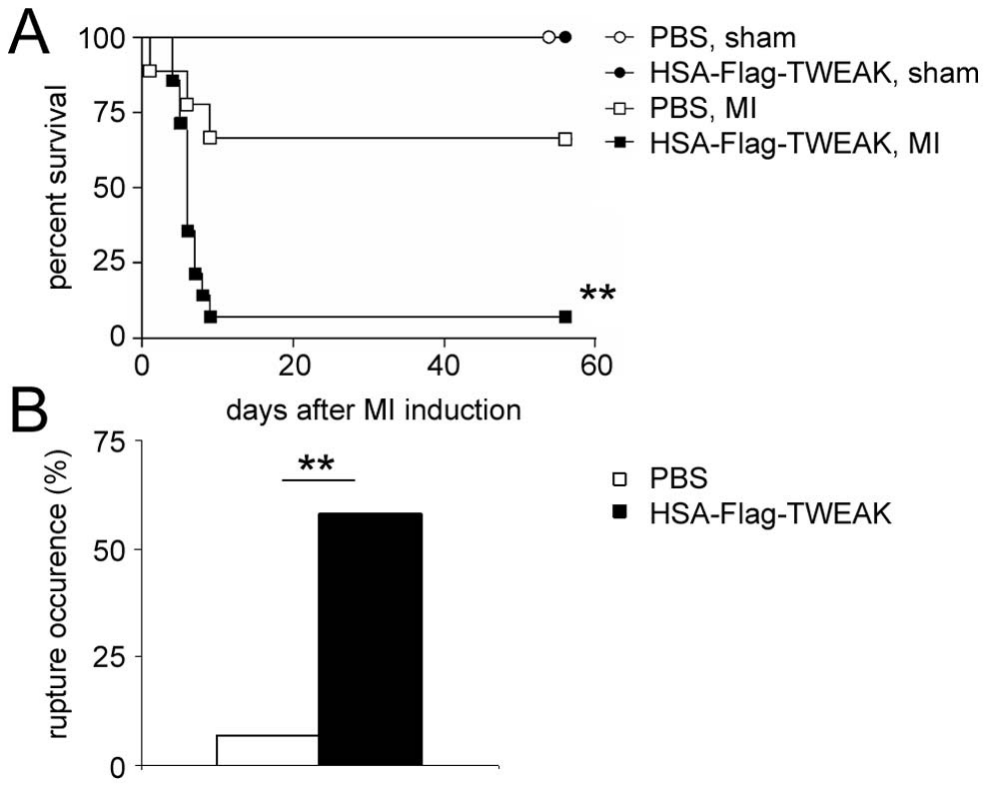
HSA-Flag-TWEAK increases mortality and cardiac ruptures after MI. (A) Percent survival was significantly deceased in HSA-Flag-TWEAK treated mice after MI compared to placebo-treated mice. (B) Most HSA-Flag-TWEAK treated mice died because of left ventricular ruptures.

Treatment of mice with HSA-Flag-TWEAK resulted in significantly lower survival rates when compared to placebo treated mice (figure 2A) without apparent changes in cardiac diameters (table 1), relative body weight, or relative weights of individual organs, namely heart, right ventricle, lung, and spleen (data not shown). The increase in mortality was mainly due to left ventricular ruptures (figure 2B); other reasons for death could not be identified in HSA-Flag-TWEAK treated mice. Thus, exogenous administration of HSA-Flag-TWEAK causes early death of infarcted mice mainly due to cardiac ruptures.

**Table 1:** HSA-Flag-TWEAK does not affect echocardiographic measurements after MI.

Parameter	PBS, sham	HSA-Flag-TWEAK, sham	PBS, MI	HSA-Flag-TWEAK, MI
**Day 1**				
**n**	3	3	9	17
**Heart rate (bpm)**	473±19	440±15	462±29	495±16
**Papillary**				
** ESA (mm^2^)**	7±0.4	5.7±0.3	12.2±0.7	11.7±0.5
** EDA (mm^2^)**	10±0.8	7.6±0.4	14.5±0.5	14.2±0.6
** 2D FS (%)**	29.6±3.0	25±0	16.1±2.5	17.4±1.5
**Apical**				
** ESA (mm^2^)**	7.6±0.4	7±1.0	13.5±0.8	11.9±0.7
** EDA (mm^2^)**	11.2±0.7	9.9±0.5	15.7±0.9	14.1±0.7
** 2D FS (%)**	32.3±4.9	29.8±6.4	14.1±1.7	15.7±1.8
**Day 3**				
**N**	3	3	7	15
**Heart rate (bpm)**	473±3	480±47	563±23	561±11
**Papillary**				
** ESA (mm^2^)**	8±1.15	6.4±0.8	12.9±1.3	12.7±0.8
** EDA (mm^2^)**	10.3±1.45	8.7±1.2	15.4±1.3	15±0.8
** 2D FS (%)**	22.7±1.46	25.3±3.1	17.3±2.0	15.8±1.2
**Apical**				
** ESA (mm^2^)**	7.6±0.29	8.2±1.4	12.8±1.4	12.7±0.8
** EDA (mm^2^)**	10.8±0.62	10.9±1.7	15.4±1.56	15±0.8
** 2D FS (%)**	29.7±2.18	25±2.2	17.1±1.6	16.5±1.8

Animals underwent echocardiography on day 1, day 3, day 21 (data not shown) and day 56 (data not shown) after MI. All measurements were recorded at the midpapillary level which shows changes in the dimensions of the surviving non-infarcted myocardium, as well as on the apical level depicting changes in scar formation.

Data are means ± sem; *n* indicates number of animals studied. EDA, end-diastolic area; ESA, end-systolic area; FS, fractional shortening; 2D, 2-dimensional.

### Extracellular Matrix Remodelling and HSA-Flag-TWEAK Treatment

Different infarct sizes could explain an increase in left ventricular rupture. However, after 6 hours of MI, no differences in infarct size, measured by Evans blue/TTC staining could be found in HSA-Flag-TWEAK treated mice in comparison to control mice (infarct size/area at risk, placebo vs. HSA-Flag-TWEAK, 48.1±6% vs. 61.2±7%, p = n.s.). Moreover, changes in the cardiac architecture after MI could be responsible for the phenotype. Yet, echocardiography demonstrated that left ventricular end-systolic (ESA) and end-diastolic area (EDA), as well as fractional shorting (FS) were not significantly altered between the groups (table 1).

**Figure 3:**
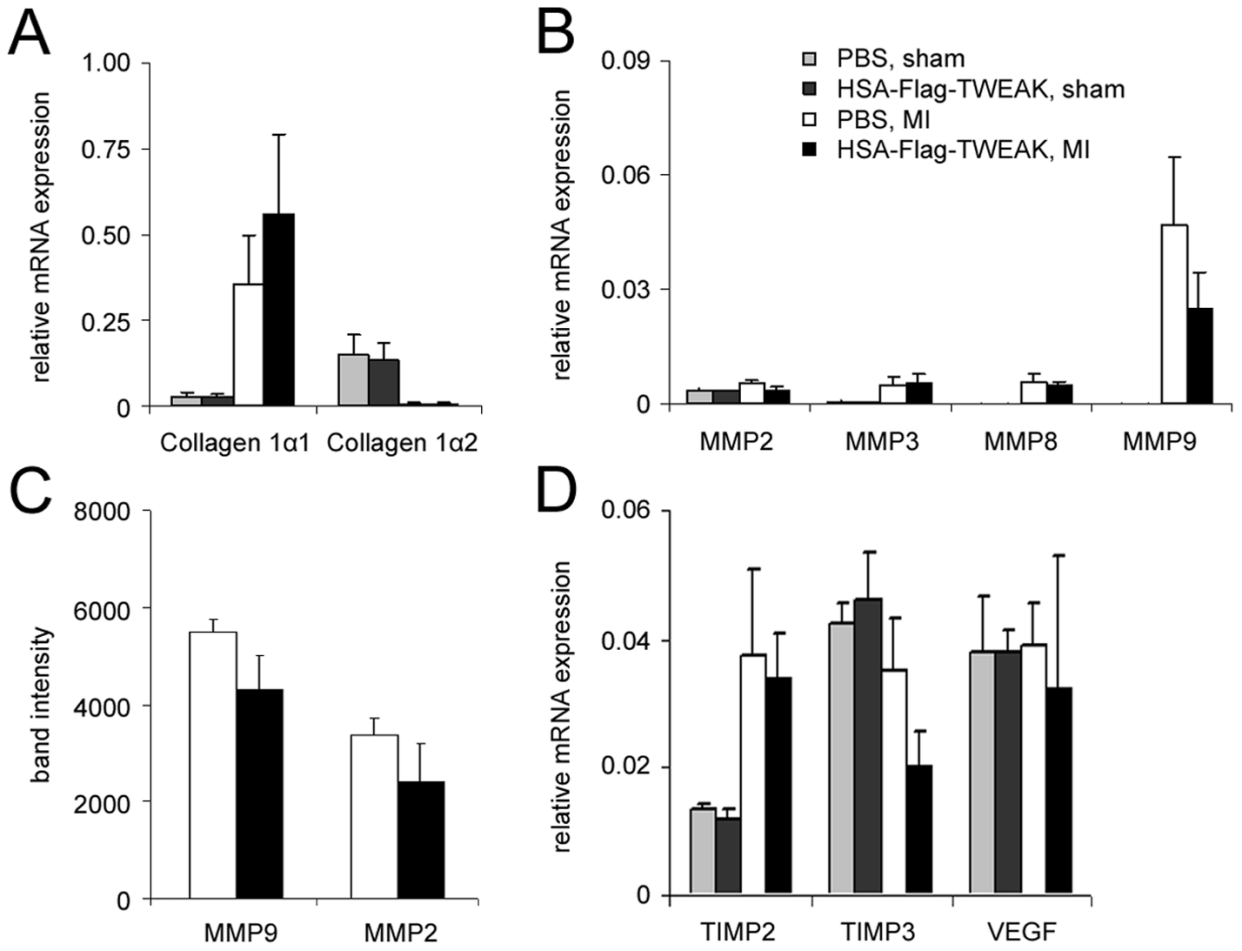
HSA-Flag-TWEAK fails to modulate extracellular matrix remodeling after MI. mRNA-expression of (A) collagen1α1, collagen1α2, (B) MMP-2, MMP-3, MMP-8, and MMP-9 were unaffected in the scar region of HSA-Flag-TWEAK challenged mice as were the (C) zymographic activities of MMP-2 and MMP-9 (measured as gel band intensity) and (D) TIMP-2, TIMP-3, and VEGF mRNA expression.

Since extracellular matrix remodeling has a pivotal role for healing after MI, we investigated components of extracellular matrix remodeling 3 days after MI, i.e. before cardiac ruptures occurred. Markers of extracellular matrix remodelling were not compromised on mRNA levels in HSA-Flag-TWEAK animals: In the infarcted area expressions of collagen1α1, collagen1α2, and matrix metalloproteinases (MMPs) 2, 3, 8, and 9 were not significantly altered (figure 3A, figure 3B). The activities of MMP-2 and MMP-9 as assessed by gelatine zymography were not different between the two treatment groups (figure 3C). The mRNA expressions of Tissue MMP inhibitors (TIMP) 2 and 3 were not different 3 days after MI, neither were the expression levels of vascular endothelial growth factor (VEGF) changed in HSA-Flag-TWEAK treated mice (figure 3D). Thus, differences in extracelluar matrix remodeling, left ventricular architecture or infarct size cannot explain our phenotype.

### Role of HSA-Flag-TWEAK for Apoptosis after MI

**Figure 4:**
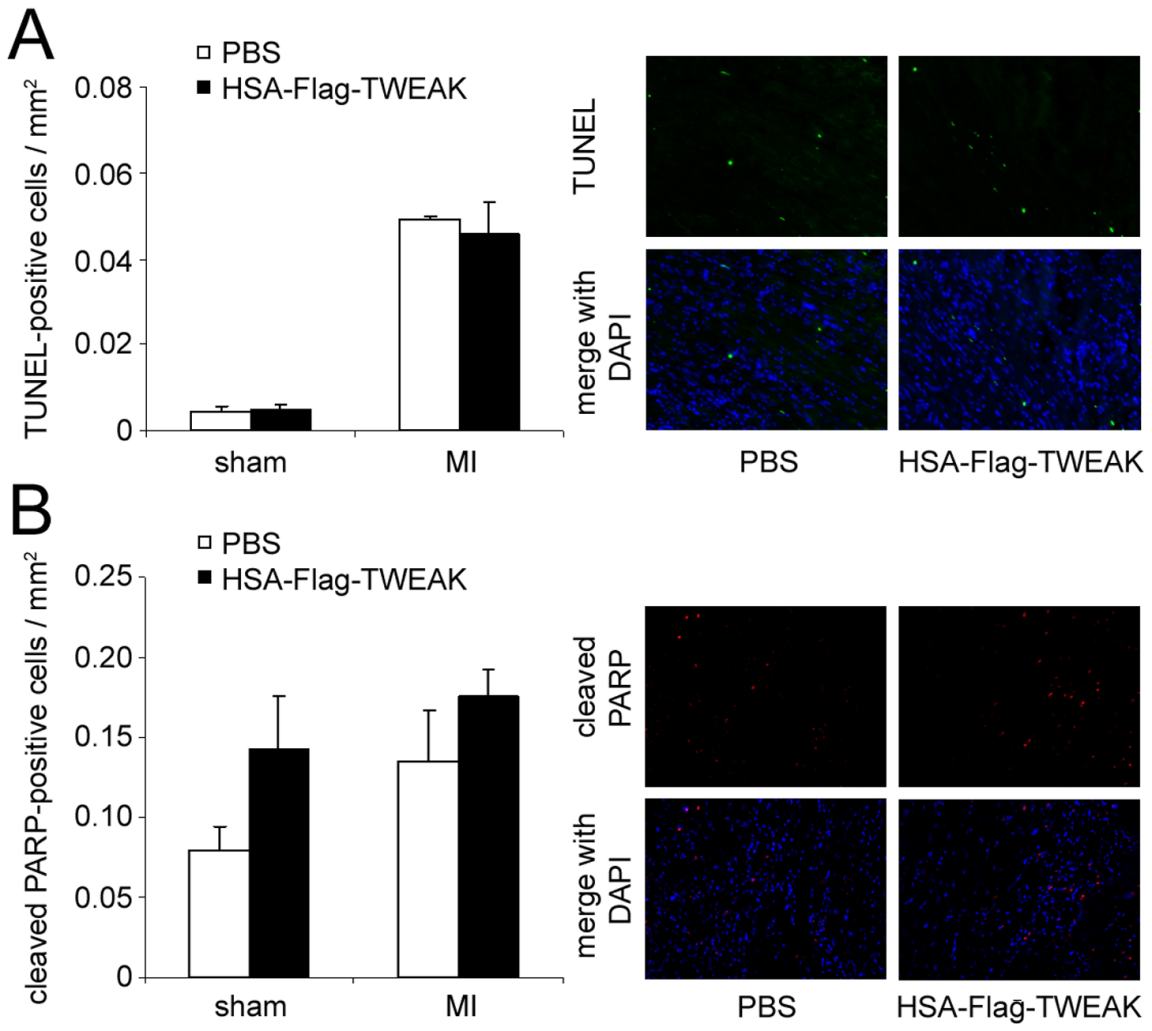
HSA-Flag-TWEAK does not induce cardiomyocyte apoptosis. The percentage of apoptotic cells 3(A) TUNEL-positive cells and (B) cleaved PARP was similar between HSA-Flag-TWEAK and PBS treated mice.

We next tested the hypothesis that apoptosis of cardiomyocytes may have contributed to the left ventricular rupture. Assessment of LV by TUNEL staining and immunohistological staining for cleaved PARP suggested that HSA-Flag-TWEAK treatment did not alter the rate of apoptosis in either MI or sham-operated mice, measured with TUNEL-staining (figure 4A) and immunohistological staining for cleaved PARP (figure 4B).

### Cytokine Array Screening and Bioinformatics Analysis Detected an Accentuation of Inflammation by HSA-Flag-TWEAK Treatment

**Figure 5:**
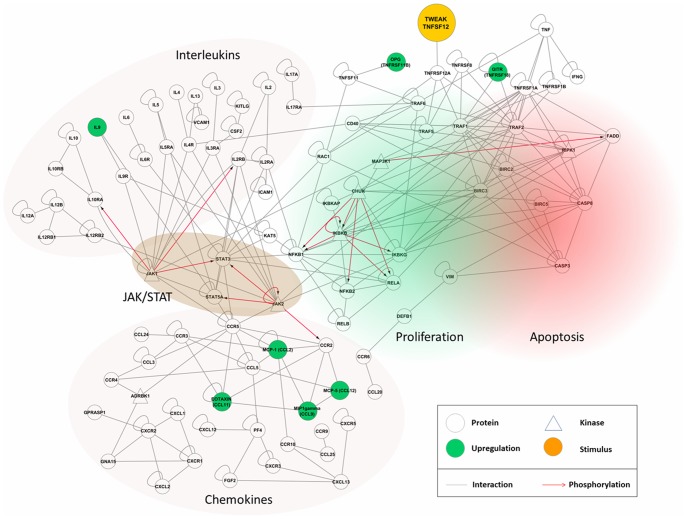
HSA-Flag-TWEAK treatment modulates the expression of cytokines and chemokines via NFκB and JAK/STAT-signalling. The protein-protein interaction network maps cytokine and chemokine data from the protein microarrays onto the human interactome. Circles indicate proteins; kinases are depicted in triangular shape, each specified by gene names. They are connected either by gray lines indicating protein-protein interactions or red arrows denoting phosphorylation reactions. The functional entities are highlighted (interleukins, chemokines, JAK/STAT pathway). Green-colored nodes show up-regulation in the TWEAK stimulation (orange) condition.

Although HSA-Flag-TWEAK has a lower inflammatory activity than membrane TWEAK or TNF, it nevertheless stimulates at low levels the classical NFκB pathway and also strongly triggers the alternative NFκB pathway which by cell type-specific mechanisms can crosstalk into the classical NFκB pathway. We thus used protein microarray technology to study alterations in the cytokine expression in the infarcted area 3 days after MI with HSA-Flag-TWEAK treatment. To better understand their functional significance we mapped the protein microarray data bioinformatically by ANOVA after normalization on the human interactome (figure 5).

The protein levels for a number of potential mediators of an exaggerated immune response were increased following *in vivo* treatment with HSA-Flag-TWEAK: Interferon-γ (IFN-γ), interleukin-12 (IL-12), glucocorticoid induced tumor necrosis factor receptor family related gene (GITR), monocyte chemotactic protein-1/-5 (MCP-1/-5), regulated and normal T cell expressed and secreted (RANTES; CCL5) and IL-5 (table 2). Functional clusters indicate interleukin and chemokine pathways and entities, connected by the JAK/STAT pathway. In conclusion, the protein array data together with bioinformatical modeling suggested a role of TWEAK stimulation in inflammatory cell activation.

**Table 2:** HSA-Flag-TWEAK modulates the production of cytokines in the infarcted heart after MI.

	PBS		HSA-Flag-TWEAK		
abbreviation/gene name	expression [pg/ml]	SEM	expression [pg/ml]	SEM	p
BLC/B-lymphocyte chemoattractant	116,18	5,54	110,40	4,81	N.S.
CD40	10,18	0,64	12,82	1,35	N.S.
CXCL16/Chemokine (C-X-C motif) ligand 16	0,50	0,30	0,43	0,43	N.S.
Eotaxin	10,52	0,31	11,63	0,36	N.S.
Eotaxin-2	15,26	0,43	16,09	0,52	N.S.
E-Selectin	40,71	2,92	45,90	5,04	N.S.
GITR/glucocorticoid induced tumornecrosis factor receptor family related gene	64,45	10,41	99,69	4,95	p<0.05
GM-CSF/Granulocyte macrophagecolony-stimulating factor	2,54	1,19	2,70	1,61	N.S.
ICAM-1	420,03	107,50	365,54	186,63	N.S.
IFN-γ	u.d.		7,47	5,75	p<0.05
IL-12/Interleukin-12	u.d.		44,51	3,23	p<0.05
IL-12p40/70	90,43	40,35	73,85	45,21	N.S.
IL-2/Interleukin-2	6,08	1,65	6,22	1,91	N.S.
IL-3/Interleukin-3	0,27	0,26	1,04	0,77	N.S.
IL-5/Interleukin-5	4,13	2,66	18,75	5,05	p<0.05
IL-9/Interleukin-9	241,45	16,66	291,12	16,77	N.S.
KC/keratinocyte-derived chemokine	1,29	0,72	2,89	0,58	N.S.
MCP-1/monocyte chemotactic protein-1	u.d.		21,54	7,27	p<0.05
MCP-5/monocyte chemoattractant protein-5	u.d.		7,36	3,65	p<0.05
MDC/macrophage-derived chemokine	19,18	1,99	22,49	1,54	N.S.
MIP-1γ/macrophage inflammatory protein γ	412,09	117,66	747,38	107,42	N.S.
MIP-2/macrophage inflammatory protein-2	1,41	0,88	7,83	3,22	N.S.
MIP-3α/macrophage inflammatory protein-3α	7,49	0,36	7,67	0,39	N.S.
OPG/Osteoprotegerin	39,80	2,60	49,27	3,46	N.S.
Osteopontin	17780,54	5216,12	22152,46	4037,31	N.S.
PF4/platelet factor 4	785,01	69,99	1362,01	337,63	N.S.
P-Selectin	363,30	40,92	551,42	105,86	N.S.
RANTES/regulated on activation, normalT cell expressed and secreted	u.d.		4,63	3,34	p<0.05
Resistin	34,46	5,18	43,76	16,07	N.S.
sTNFRII	14,77	0,94	17,12	1,38	N.S.
VCAM-1/vascular cell adhesion molecule 1	168,49	23,01	257,27	43,26	N.S.
VEGF-D/vascular endothelial growth factor D	91,54	12,95	84,96	5,79	N.S.

u.d. = under detection limit.

IFN-γ: <11 pg/ml; IL-12: <5 pg/ml; MCP-1: <5 pg/ml; MCP-5: <1 pg/ml; RANTES: <3 pg/ml.

A cytokine array for the detection of the protein expression of different cytokines was performed 3 days after MI. HSA-Flag-TWEAK treated mice showed statistically significant up-regulation of interleukin 12 (IL-12), interleukin 5 (IL-5), monocyte chemotactic protein-1 (MCP-1), macrophage inflammatory protein-2 (MIP-2) and regulated on activation, normal T cell expressed and secreted (RANTES) (u.d. = under limit of detection).

### Immune Cells are Involved in HSA-Flag-TWEAK Mediated Effects

**Figure 6:**
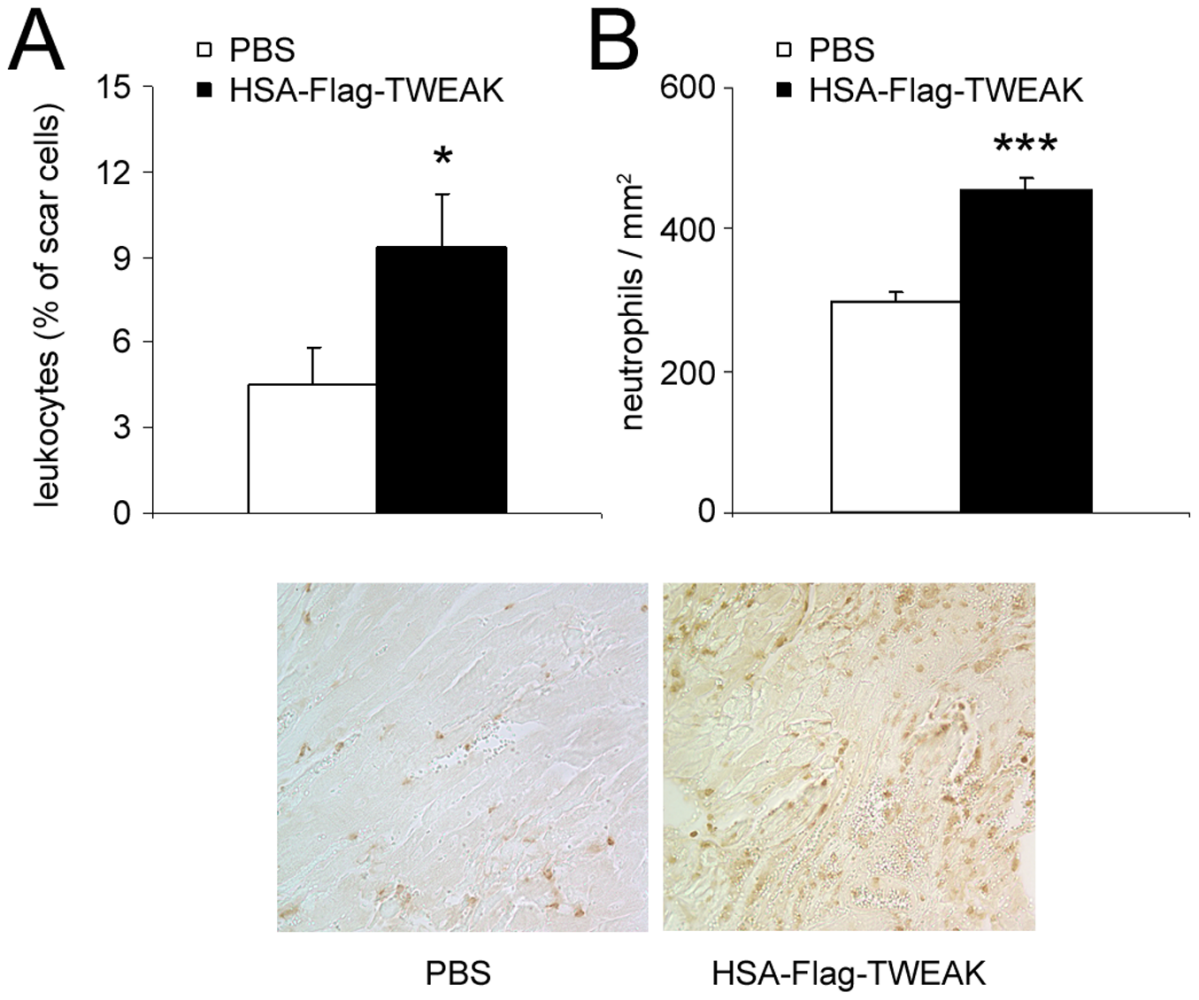
HSA-Flag-TWEAK increases immune cell infiltration into the infarcted heart. (A) Exogenous administration of HSA-Flag-TWEAK amplified the infiltration of leukocytes into the infarcted myocardium 3 days after MI as determined by FACS analysis. (B) Neutrophils highly infiltrated the border zone of HSA-Flag-TWEAK treated mice.

The microarray/bioinformatical results also indicated the potential involvement of immune cells in the detrimental effects of HSA-Flag-TWEAK. Therefore, we next analyzed the content of different immune cell subsets in mice hearts after MI by FACS-analysis. We found significantly more CD45^+^ cells infiltrating the infarcted area of HSA-Flag-TWEAK in comparison to placebo treated mice (figure 6A). Histological staining proved increased infiltration of neutrophils into the border zone of mice treated with HSA-Flag-TWEAK (figure 6B).

**Figure 7:**
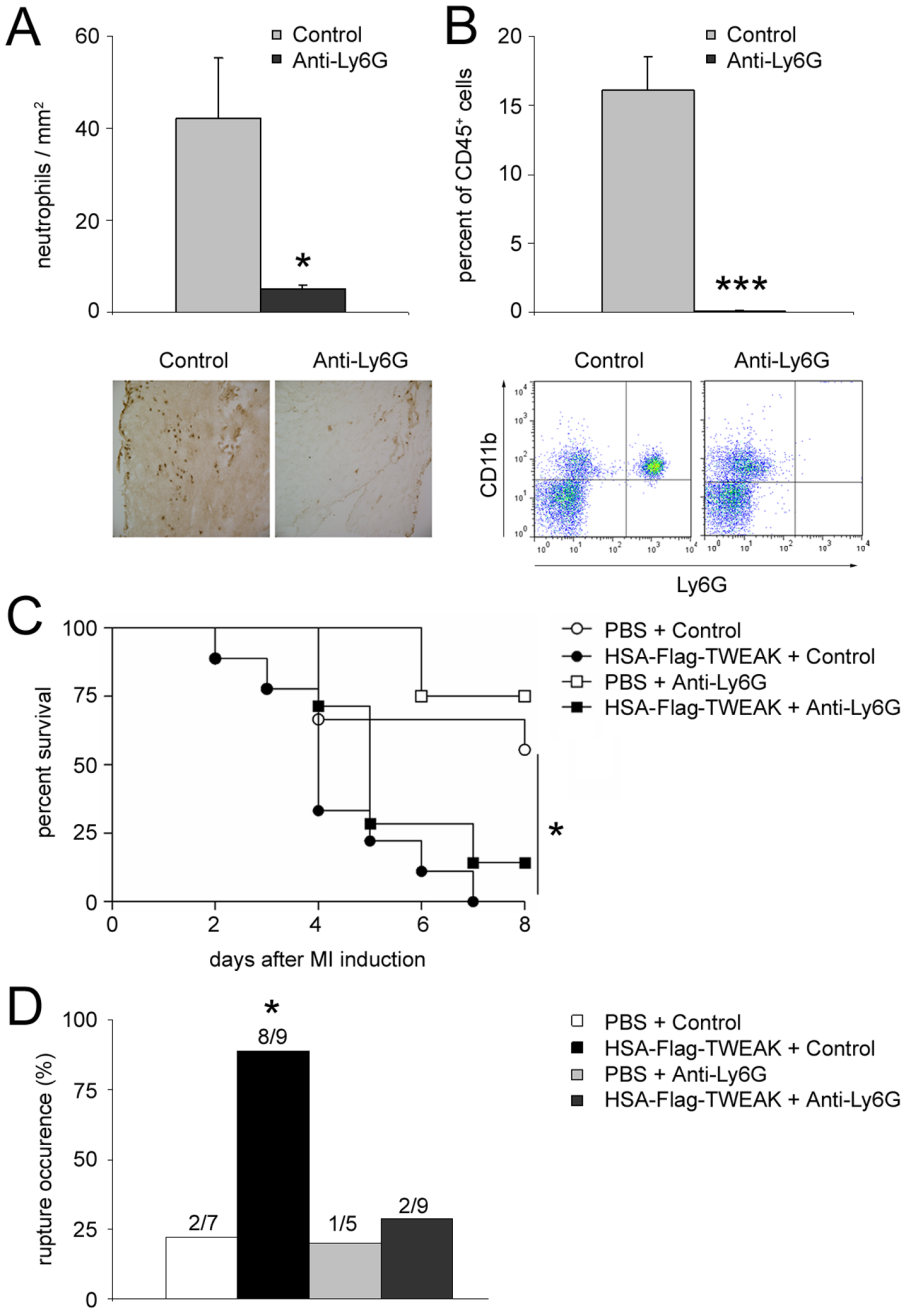
The induction of cardiac ruptures by HSA-Flag-TWEAK depends on neutrophils. Mice were treated with an anti-Ly6G antibody to deplete neutrophils (A) in the myocardium as measured by immunohistological staining and (B) in the peripheral blood as measured by FACS analysis. (C) Neutrophil depletion did not affect survival after MI in PBS and HSA-Flag-TWEAK treated mice. (D) The occurrence of cardiac ruptures was significantly reduced after anti-Ly6G antibody treatment in the HSA-Flag-TWEAK treated group in comparison to the neutrophil intact control.

Several publications describe a correlation between the occurrence of cardiac ruptures and neutrophil infiltration [28], [29]. To test whether the HSA-Flag-TWEAK-mediated increase in neutrophil infiltration is responsible for cardiac ruptures, we depleted neutrophils in mice *in vivo* by treating them with anti-Ly6G antibodies after HSA-Flag-TWEAK application. Effective depletion of neutrophils was confirmed in heart tissue slices (figure 7A) and by flow cytometry in peripheral blood samples using CD11b^+^Ly6G^+^ as a percent of CD45^+^ cells (figure 7B).

Treatment with HSA-Flag-TWEAK resulted in significantly increased mortality in neutrophil intact mice (figure 7C). Neutrophil depletion in HSA-Flag-TWEAK challenged mice did not modify MI-induced mortality (figure 7C), but significantly reduced the occurrence of cardiac ruptures in comparison to the placebo groups (figure 7D).

Whereas systemic neutrophil depletion did not prevent the increased mortality of HSA-Flag-TWEAK treated mice after chronic MI, the depletion of neutrophils significantly decreased the occurrence of cardiac ruptures in HSA-Flag-TWEAK treated mice.

## Discussion

Inflammation is an important contributor to heart failure [30], [31]. The goal of this study was to find an immunomodulatory therapy to improve cardiac repair and LV remodelling after MI. We tested the effects of exogenous administration of HSA-Flag-TWEAK, a recombinant variant of the naturally occurring soluble form of the multi-functional cytokine TWEAK which, together with its receptor Fn14, is robustly up-regulated after MI and can influence cardiac repair [32], ischemic tolerance [12], inflammatory processes, and apoptosis [9]. We hypothesized that this TWEAK variant would beneficially influence wound healing after MI; however, our experiments demonstrated that administration of the variant in a model of non-reperfused infarction resulted in high mortality and an increased incidence of rupture.

There are several potential mechanisms for this finding that have to be taken into account:

### Non-reperfused Infarction

We used a MI model with a non-reperfused infarction. It is likely that, at least for several days and until neovessels are formed the agent TWEAK may be only delivered to the border zone and to the non-infarcted myocardium. Findings may have been different in models of reperfused infarction.

### Changes in Extracellular Matrix Remodelling

Extracellular matrix deposition and organization play a major role in LV remodeling, and both collagen and collagen degrading enzymes are important contributors to the development of cardiac ruptures [33]. A loss of collagen struts which tether cardiomyocytes to each other can cause lengthening of infarcted cardiomyocytes under continuous stretch. This leads to infarct expansion and can provoke cardiac ruptures [34]. Thus, one potential mechanism for impaired healing and the occurrence of cardiac ruptures in HSA-Flag-TWEAK treated mice could be an imbalance of collagen content in the scar region, e.g. inadequate collagen production in the scar area or perturbed expression of collagen degrading enzymes or their inhibitors. We could show, that fibroblasts, which are the main sources for collagen, highly express Fn14 and can therefore respond to TWEAK. Nevertheless, we could not observe any effect of HSA-Flag-TWEAK on either the expression of collagen, MMPs or TIMPs, nor on the activity of MMPs in mice after MI.

### Changes in Inflammation

Inflammation is another major player in myocardial ruptures. We could recently show that CD4^+^ T cells are of importance in wound healing after MI and deficiency in these cells leads to early mortality with a high incidence of cardiac ruptures [30]. Other immune cells like macrophages and neutrophils can also influence wound healing after MI. Depleting monocytes and macrophages with clodronate-containing liposomes increases mortality in mice after MI due to thromboembolic events [35]. The depletion of neutrophils in dogs [36] or treatment with inhibitors of neutrophil adhesion in pigs [37] led to a significant decrease in infarct size suggesting that neutrophils are involved in myocardial injury.

TWEAK exhibits pro-inflammatory properties while genetic ablation of TWEAK or the application of TWEAK-blocking antibodies reduces inflammation and disease severity in TNBS-induced colitis [38]. We could show HSA-Flag-TWEAK to upregulate a number of cytokines which share the common feature of controlling trafficking and activation of innate immune cells: IFN-γ [39], MCP-1 [40] and RANTES [41]:

IFN-γ can control trafficking, activation and apoptosis of polymorphonuclear neutrophils (PMN) [39]. Furthermore, PMN were shown to produce and to release IFN-γ in response to IL-12 [42] and increased levels of this cytokine can lead to reduced wound strength [43]. IFN-γ can induce left ventricular dilation and impaired systolic function as was shown with mice overexpressing this cytokine [44].

MCP-1 mediates the recruitment of monocytes, lymphocytes [45], and neutrophils [40]. In patients, elevated MCP-1 levels are associated with attenuated remodeling during the first few days after MI. In the sub-acute phase of infarction, a rise in MCP-1 is associated with progressive adverse ventricular remodeling [46].

HSA-Flag-TWEAK treatment also up-regulated tissue levels of RANTES, which is produced by endothelial cells [47] and lymphocytes [48] and acts as a potent chemoattractant for monocytes [41], NK cells [49], and neutrophils [50] during inflammation. Treatment with RANTES antagonists decreased reperfusion injury in atherosclerotic mice [51]. This cytokine interacts with CCR1, CCR3, and CCR5. CCR1-deficiency, but not CCR5-deficiency affects the infarct size after MI in mice, whereas the latter was shown to be important for LV remodelling [52], [53].

In good accordance with the high expression of the above mentioned cytokines, we found significantly increased numbers of infiltrating CD45^+^ immune cells within the infarcted area of hearts from mice challenged with HSA-Flag-TWEAK. Among these cells, especially neutrophils were increased, indicating that TWEAK promotes the recruitment or trafficking of these cells to sites of inflammation. A good correlation between neutrophil infiltration and the occurrence of cardiac ruptures was demonstrated in patients after MI [28]. Haile et *al.* showed TWEAK to induces the passage of neutrophils to the abluminal side in an *in vitro* model of the blood-brain barrier [54] and TNF was also shown to control the recruitment of neutrophils in immune complex peritonitis [55] and during airway inflammation [56]. Therefore, our data imply that the TWEAK-induced recruitment of neutrophils to the infarcted myocardium might be mediated by NFκB dependent production of IFN-γ, MCP-1 and RANTES.

### Functional Involvement of Neutrophils in Cardiac Ruptures

To functionally test whether neutrophils are the underlying cause of HSA-Flag-TWEAK-induced cardiac ruptures, we depleted neutrophils prior to MI. This significantly reduced the incidence of ruptures without affecting HSA-Flag-TWEAK-induced global mortality. Since the mortality in mice treated with HSA-Flag-TWEAK was not affected by neutrophil depletion despite a marked reduction in the incidence of cardiac ruptures, we propose that additional mechanisms must be involved in TWEAK-induced mortality. Besides the cytokines MCP-1/-5 and RANTES, IFN-γ, IL-12 and IL-5 were highly upregulated 3 days after MI in HSA-Flag-TWEAK treated mice. The elevated levels of these cytokines suggest an involvement of other immune cells in HSA-Flag-TWEAK induced mortality after MI.

In summary, we demonstrate that treatment of mice with recombinant HSA-Flag-TWEAK after MI results in cardiac ruptures in a neutrophil-dependent manner. These results demonstrate the interplay between TWEAK, leukocytes, and cardiac wound healing after MI.
